# A novel technique of ultrasound-guided nerve root block: anterior compression lateral approach

**DOI:** 10.1007/s10396-025-01588-3

**Published:** 2025-10-18

**Authors:** Naofumi Hashiguchi, Yasushi Fujiwara, Nanoha Sato, Akiko Matsumoto, Yasushi Murakami, Shinji Kotaka, Ryo Ota, Nobuo Adachi

**Affiliations:** 1 Present Address: Department of Orthopaedic Surgery, Graduate School of Biomedical and Health Sciences, Hiroshima University, 3-2-1 Kasumi, Minami-Ku, Hiroshima City, Hiroshima 734-0037 Japan; 2 Department of Orthopaedic Surgery/Microscopic Spine and Spinal Cord Center, Hiroshima City North Medical Center Asa Citizens Hospital, 1-2-1 Kameyama Minami, Asakita-Ku, Hiroshima City, Hiroshima 731-0293 Japan

**Keywords:** Cervical radiculopathy, Ultrasound-guided nerve block, Anterior compression lateral ultrasound-guided approach, Fluoroscopy, Injectate distribution

## Abstract

**Purpose:**

Conventional posterior ultrasound-guided selective cervical nerve root block (SNRB) often fails to deliver injectate reliably into the neural foramen, while fluoroscopic guidance involves radiation exposure and specialized equipment. We developed a novel anterior compression lateral (ACL) ultrasound-guided approach to provide radiation-free, real-time visualization with improved intraforaminal delivery. This study compared ACL with conventional ultrasound (US) and fluoroscopy (FL) in terms of needle placement accuracy and injectate distribution.

**Methods:**

This retrospective single-center cohort study measured needle tip distance from the lateral mass on anteroposterior radiographs. Contrast distribution was classified as foraminal, junctional, or extraforaminal on radiographs and confirmed with axial CT in the US and ACL subgroups. Craniocaudal spread distance was also quantified.

**Results:**

A total of 114 patients with cervical radiculopathy underwent SNRB using FL (*n* = 56), US (*n* = 25), or ACL (*n* = 33). Radiographic intraforaminal distribution occurred in 76.8% of FL, 72.7% of ACL, and 16.0% of US injections (*P* < 0.0001). Needle tips in US and ACL were positioned more lateral than FL (mean offsets 4.3 ± 6.8 mm and 2.5 ± 3.9 mm vs − 3.5 ± 2.6 mm, respectively). Injectate spread was greater with US (30.8 ± 9.6 mm) and ACL (25.9 ± 15.1 mm) than FL (15.9 ± 10.7 mm) (*P* < 0.0001). On CT, ACL achieved higher intraforaminal contrast than US (72.7% vs 16.0%, *P* < 0.0001). No major complications occurred.

**Conclusion:**

The ACL ultrasound-guided approach delivers intraforaminal injectate with accuracy comparable to fluoroscopy while eliminating radiation exposure. It outperforms conventional posterior ultrasound in targeting consistency and offers a precise, accessible option for outpatient cervical SNRB.

## Introduction

Cervical nerve root block is a well-established intervention for patients with radiculopathy due to cervical spondylosis [1, 2]. Traditionally, this procedure has been performed under fluoroscopic guidance to ensure precise delivery of injectate into the neural foramen [1]. While effective, this method exposes patients and staff to ionizing radiation and requires specialized facilities and training [3]. These limitations restrict its utility, particularly in outpatient or resource-limited settings [4].

Ultrasound-guided techniques have emerged as attractive alternatives, offering real-time needle visualization and elimination of radiation exposure [5]. In clinical practice, posterior approaches under ultrasound guidance are commonly used [6]. However, a previous study has shown that this technique often results in injectate accumulation near the foraminal rim rather than within the foramen itself [7]. This anatomical limitation may reduce the effectiveness of selective nerve root blockade and increase the need for repeated procedures [8].

To address this limitation, attention has shifted toward anterior or anterolateral approaches that target the foramen more directly. Several computed tomography (CT)-guided techniques using an anterolateral route with patients in the supine position have demonstrated favorable outcomes, including reliable needle placement and sustained symptom improvement [9–11]. These approaches enable safe access to the neural foramen on the compressed side by advancing the needle in a near-horizontal plane while avoiding major vascular structures under image guidance. Despite their efficacy, CT-guided techniques still require specialized imaging equipment and subject patients to radiation exposure.

Building upon the principles of CT-guided anterolateral approaches, we developed an anterior compression lateral (ACL) ultrasound-guided approach. This technique adapts the anterior transforaminal trajectory to an ultrasound-based protocol. With the patient in a supine position with the head turned contralaterally, the ultrasound probe is placed anterolaterally and used to gently displace vascular structures, creating a safe acoustic window toward the intervertebral foramen. The needle is advanced in-plane under continuous ultrasound monitoring, aiming for direct intraforaminal delivery while preserving procedural safety and reproducibility.

The purpose of this study was to evaluate whether the ACL approach offers improved needle placement accuracy and more favorable injectate distribution compared to both the conventional posterior ultrasound approach and the standard fluoroscopy-guided technique. We hypothesized that the ACL method would achieve intraforaminal spread comparable to fluoroscopy while offering the practical advantages of ultrasound guidance.

## Materials and methods

### Study design and setting

**Fig. 1:**
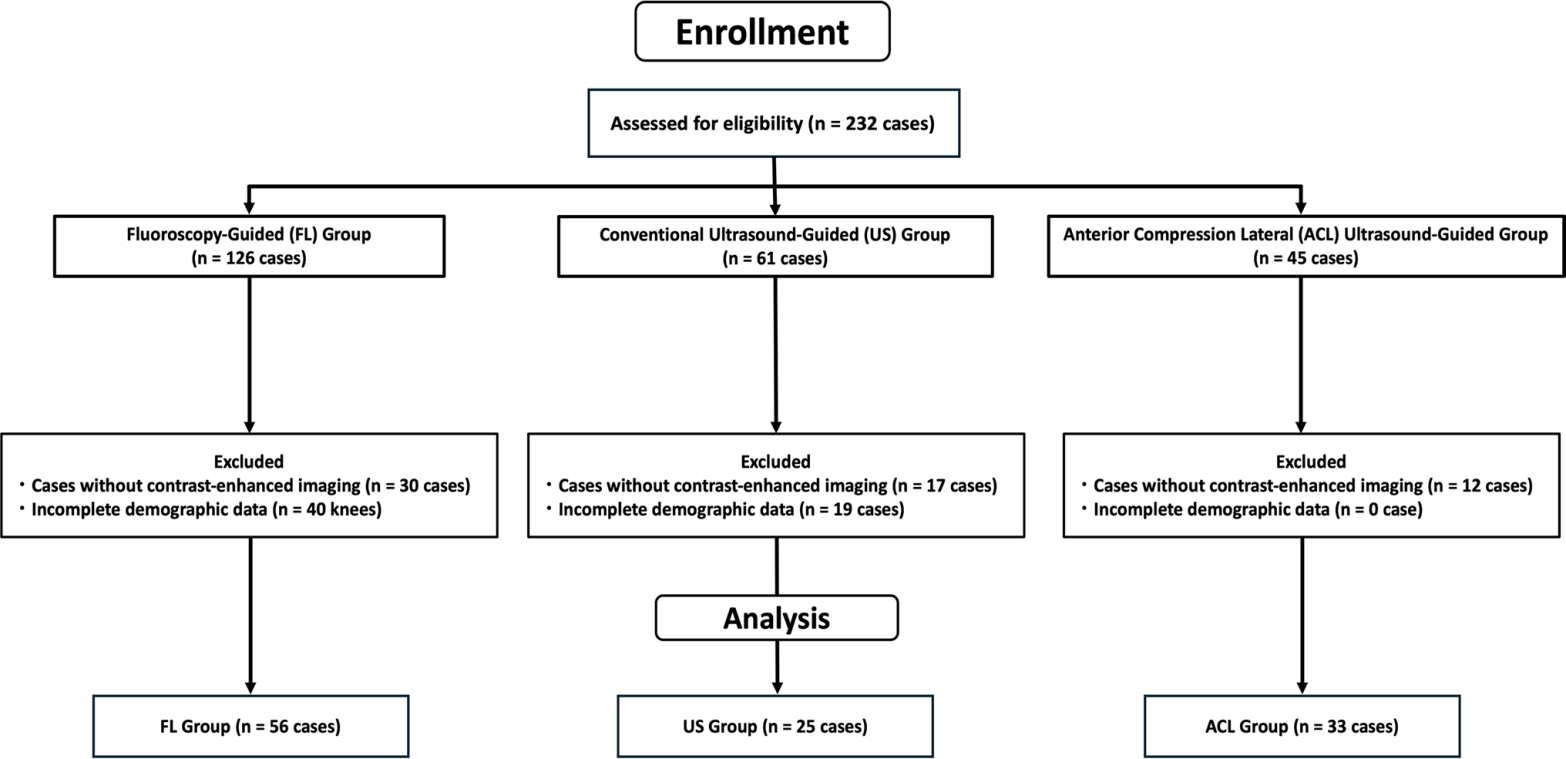
Flowchart of patient inclusion and exclusion. A total of 114 patients with cervical radiculopathy were included and categorized into three groups according to the image-guidance technique used: fluoroscopy (FL), conventional ultrasound (US), and anterior compression lateral ultrasound (ACL)

We conducted a single-center, retrospective cohort study of individuals who underwent cervical nerve root block at our institution between January 2017 and January 2020. The institutional review board approved this study (06–6-10), ensuring adherence to ethical guidelines and the Strengthening the Reporting of Observational Studies in Epidemiology (STROBE) statement for reporting observational studies [12]. We screened individuals diagnosed with cervical radiculopathy via clinical examination and magnetic resonance imaging. We excluded patients with missing anthropometric data (height, body weight, or body mass index [BMI]), coagulation disorders, active infection, anatomical anomalies, known allergy to iodinated contrast medium, or those who declined consent for contrast-enhanced imaging (Fig. 1).

### Nerve root block procedures

We divided patients into three groups according to guidance technique: fluoroscopic guidance (FL), conventional ultrasound guidance (US), and an ACL ultrasound-guided approach. Operators followed standardized protocols for each technique.

### Fluoroscopy-guided nerve root block (FL group)

Under sterile conditions, patients were positioned supine with slight neck extension [13]. A fluoroscopy unit was used to identify the target level. Three spine surgeons each with more than 15 years of experience performed the procedure. A 22-gauge cathelin needle was advanced toward the neural foramen using an oblique anterior approach under real-time biplanar fluoroscopy. These steps were taken to minimize between-group variability in magnification and acquisition conditions. We injected 0.2 mL of Omnipaque 240 contrast medium and then administered a mixture of 1 mL of 1 percent lidocaine and 1 mL containing 3.3 mg of dexamethasone.

### Ultrasound-guided nerve root block (US group)

**Fig. 2:**
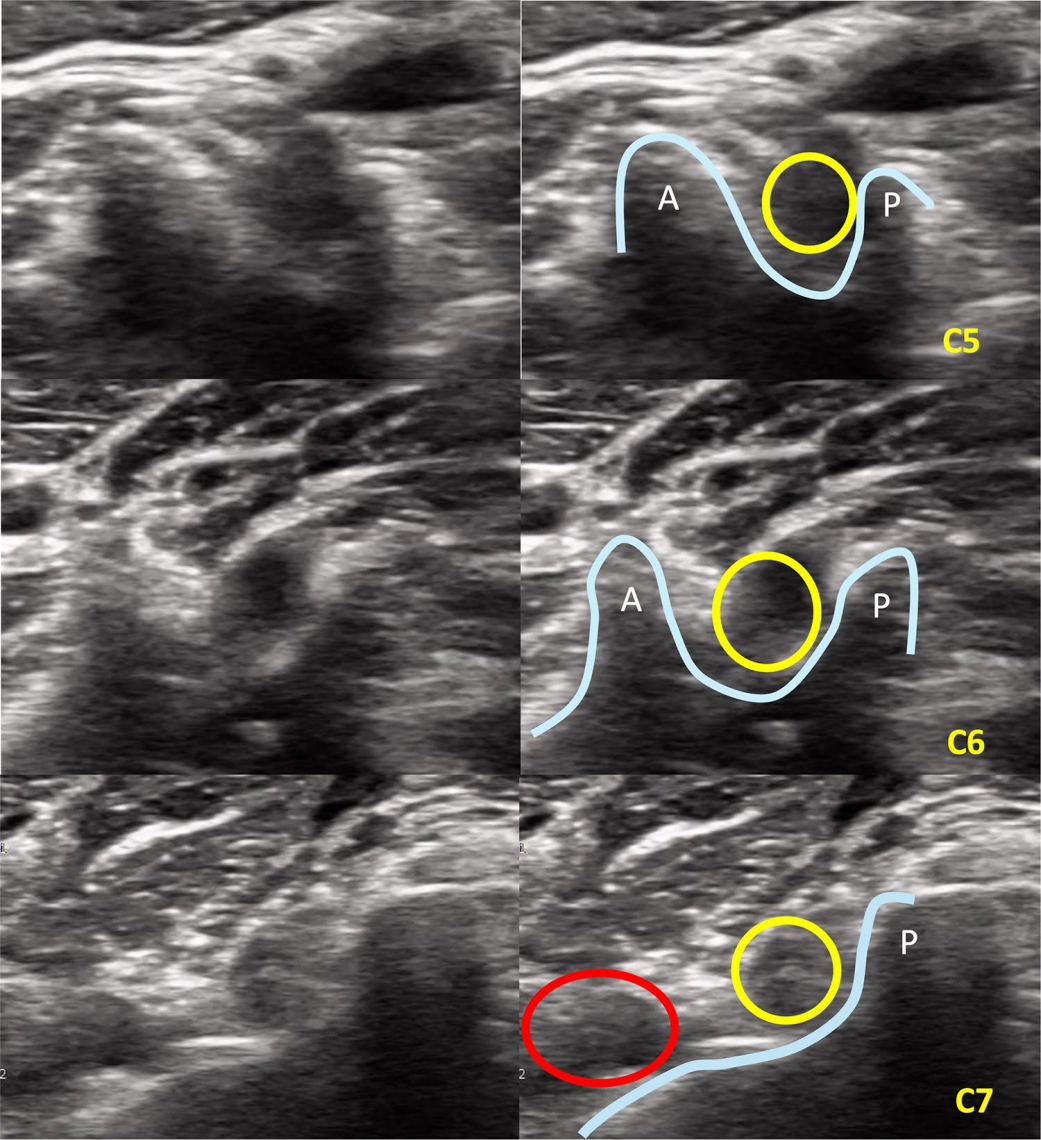
Axial ultrasound anatomy of the cervical nerve roots and surrounding structures from C5 to C7 levels. Transverse sonographic images obtained at the C5 (top), C6 (middle), and C7 (bottom) cervical levels, demonstrating anatomical landmarks relevant to the conventional US approach. Left panels show original ultrasound images; right panels include schematic overlays for orientation. Yellow circles indicate the exiting cervical nerve roots. Blue lines trace the bony contour formed by the anterior (A) and posterior (P) tubercles of the transverse processes, which serve as critical sonographic landmarks during the conventional US approach. At the C7 level, the anterior tubercle is characteristically absent or underdeveloped, resulting in a flattened anterior contour. The red circle marks the vertebral artery, which is located anterolateral to the transverse process and must be carefully avoided during needle planning

We used the SONIMAGE MX1 ultrasound system (KONICA MINOLTA, Japan) with an L11-3 transducer in an axial oblique view to identify the transverse process and exiting nerve root. We used the absence of an anterior tubercle at the seventh cervical vertebra as a landmark to identify the target level for the block [14]. All ultrasound examinations were conducted by an experienced orthopedic surgeon (NH) with 5 years of clinical ultrasound experience. We advanced the needle in‐plane from lateral to medial, aiming toward but not entering the intervertebral foramen (Fig. 2). We used the same needle and the same volume of injectate as in the FL group.

### Anterior compression lateral approach (ACL group)

**Fig. 3:**
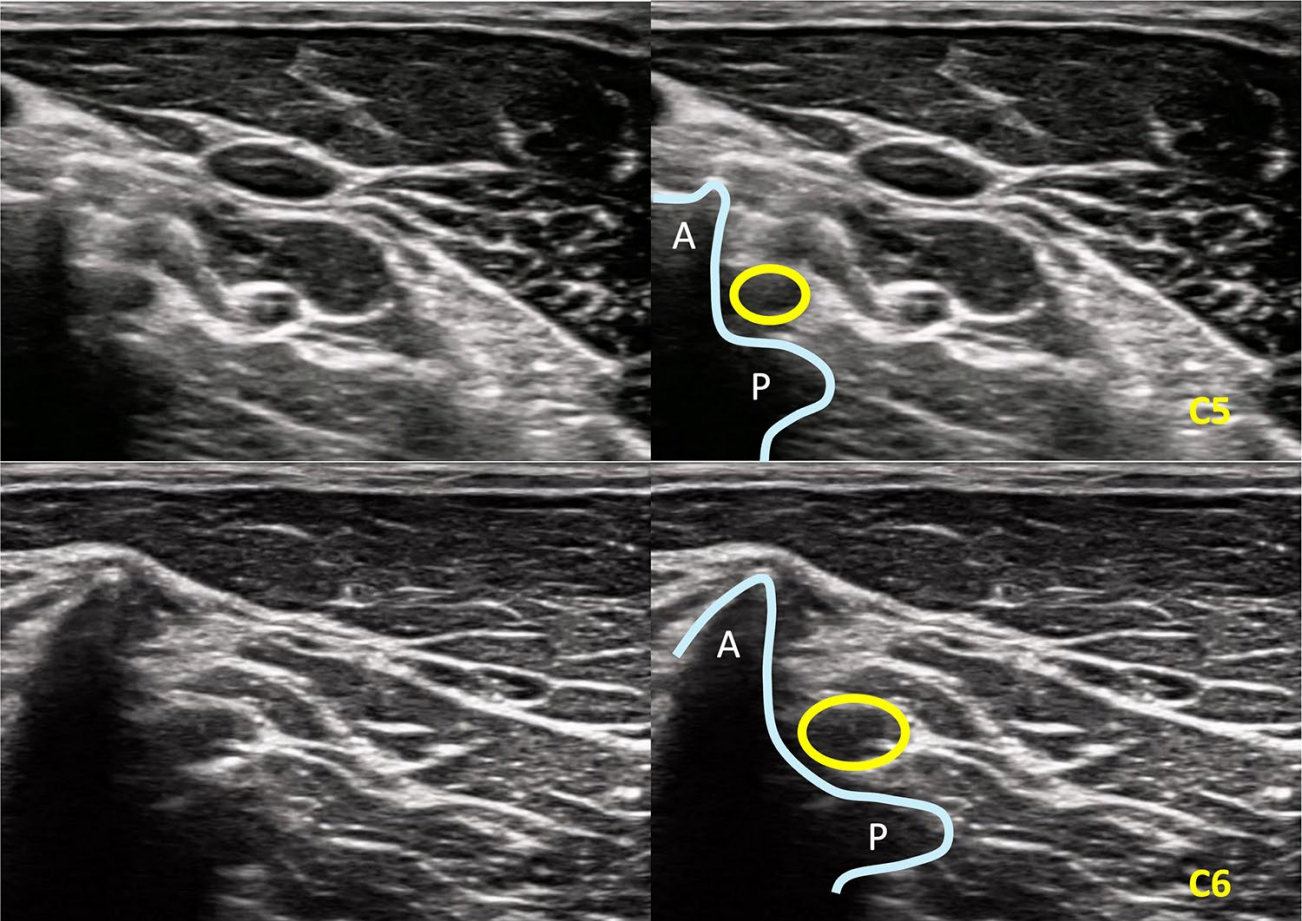
Ultrasound images of the anterior compression lateral (ACL) ultrasound-guided approach at the C5 and C6 cervical levels. Transverse sonographic images at the C5 level (top row) and C6 level (bottom row) obtained during the ACL approach. Left panels show baseline anatomical visualization of the transverse processes and cervical nerve roots. Right panels demonstrate schematic overlays for orientation. Yellow circles indicate the exiting cervical nerve roots (C5 and C6), and blue curved lines trace the bony contour formed by the anterior (A) and posterior (P) tubercles of the transverse processes. This anatomical corridor serves as a key sonographic landmark for safe planning of the needle path during the ACL technique, while avoiding vascular structures anterior to the transverse process

**Fig. 4:**
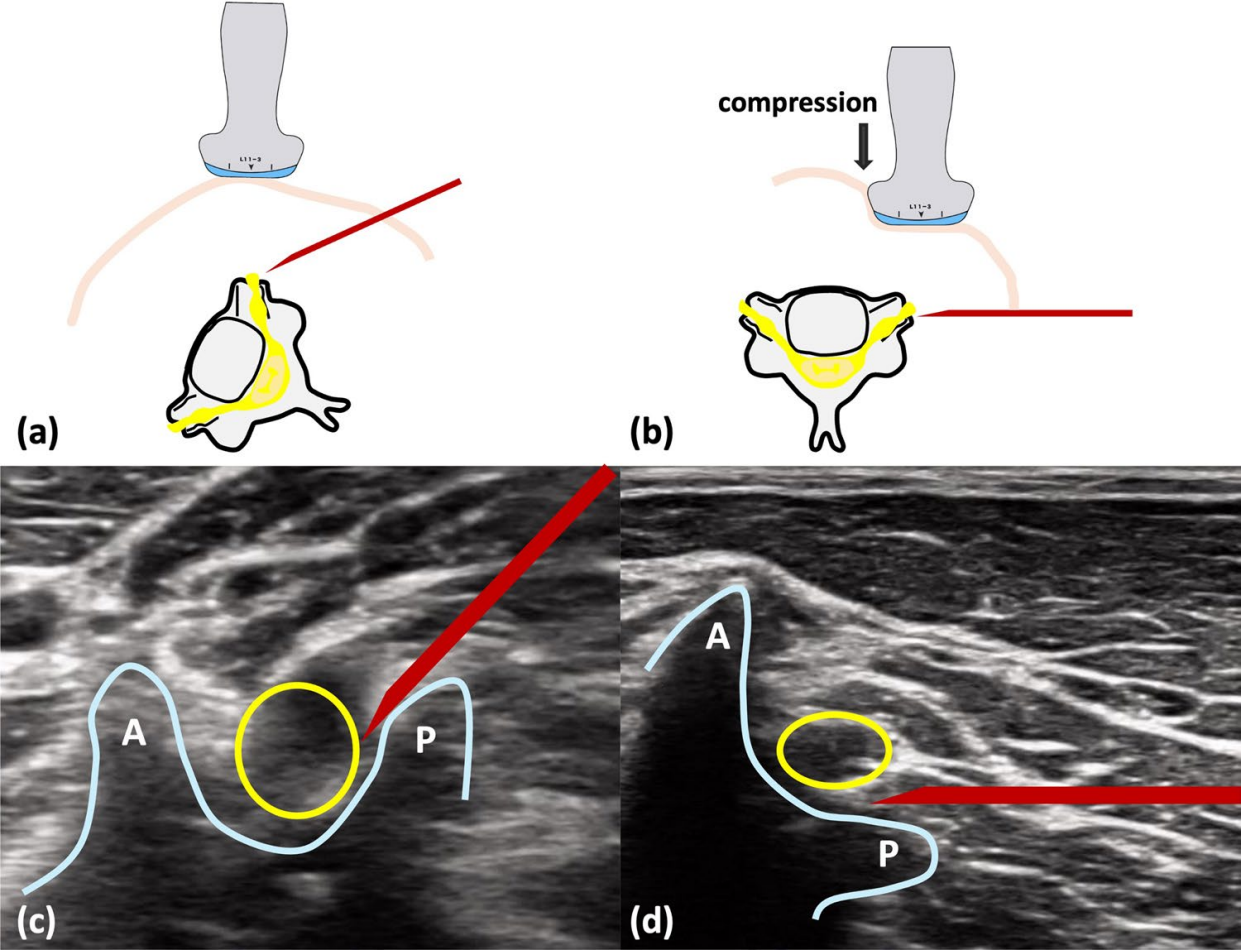
Conventional ultrasound and anterior compression lateral (ACL) ultrasound-guided approaches. **a**, **c** The patient lies in the lateral decubitus position, and the probe is placed laterally for conventional ultrasound-guided cervical selective nerve root block. **a** A schematic diagram showing the posterolateral in-plane needle trajectory advancing toward the neural foramen. **c** The corresponding ultrasound image with colored overlays indicating anterior (A) and posterior (P) tubercles of the transverse processes and the needle entering from the posterolateral side. **b**, **d** The ACL technique is depicted with the patient supine. **b** A schematic that illustrates anterior probe placement with slight medial compression to align the imaging plane parallel to the intervertebral foramen and a lateral-to-medial in-plane needle approach. **d** The real ultrasound image of the ACL approach with the same landmark cues and the needle advancing from lateral toward the target

We developed the ACL technique by adapting a CT-guided lateral approach described in prior studies [15–20]. Patients lay supine facing upward while operators aligned the ultrasound machine, probe, and planned needle path. The probe was placed anteriorly on the neck, and the operator applied slight medial compression only on the inner edge of the probe to orient the imaging plane parallel to the intervertebral foramen (Fig. 3). We identified the target nerve root level by locating the seventh cervical vertebra, which lacks an anterior tubercle [14]. We scanned the anticipated lateral-to-medial needle trajectory to confirm the absence of vascular structures or off-target nerves. After measuring the depth from skin to the planned nerve root, we advanced the needle from lateral toward medial under continuous in-plane ultrasound imaging (Fig. 4). We used the same needle and the same volume of injectate as in the FL group.

### Outcome measures: needle tip position and injectate spread distance

**Fig. 5:**
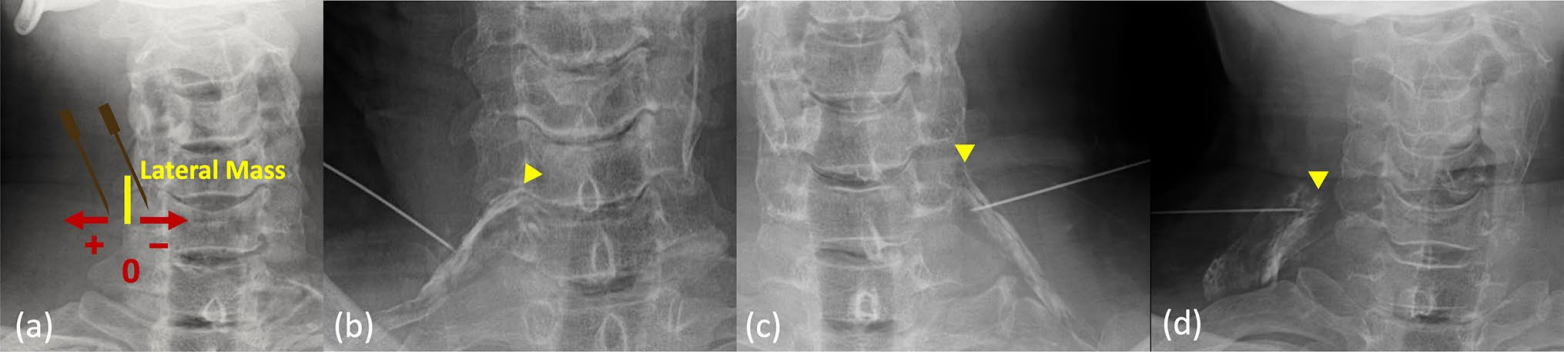
Fluoroscopic assessment of needle localization and contrast distribution. **a** An anteroposterior fluoroscopic image with schematic overlay and a reference line along the lateral margin of the lateral mass; the horizontal distance from that line to the needle tip is measured in millimeters with lateral positions recorded as positive, medial as negative, and the margin itself as zero. **b**–**d** Anteroposterior radiographs with the most proximal contrast marked by a yellow arrowhead. **b** The intraforaminal zone (F) where contrast lies entirely medial to the lateral mass. **c** The junctional zone (J) with contrast extending within 2 mm of the lateral mass. **d** The extraforaminal zone (E) where contrast extends more than 2 mm lateral to the lateral mass

Needle tip distance was measured from the lateral mass on anteroposterior imaging. This landmark was chosen for its reliability and ease of identification on X-ray, as supported by previous cadaveric and clinical studies [13, 21–23]. We measured the needle tip position relative to the lateral mass and recorded positions medial to the lateral mass as negative and lateral to it as positive (Fig. 5). After injecting 0.2–0.3 mL of contrast through the needle tip, we obtained radiographs and measured the maximum longitudinal spread distance of the injectate along the nerve root path.

### Contrast distribution

**Fig. 6:**
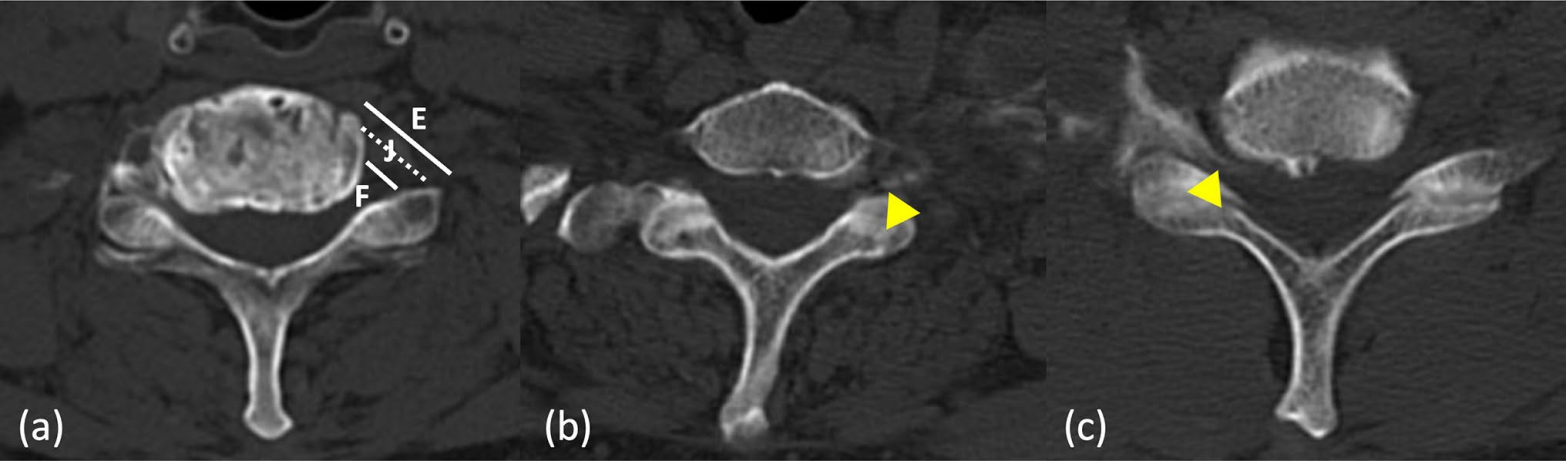
Classification of contrast distribution on axial CT images. **a** The anatomic zones used to classify contrast spread following cervical nerve root block. An oblique reference line (dashed) is drawn from the anterolateral margin of the vertebral body to the lateral margin of the facet joint. Contrast located entirely medial to this line is categorized as the foraminal zone (F). Contrast lying directly on or within 2 mm of either side of the line is classified as the junctional zone (J), and contrast extending more than 2 mm lateral to the line is considered extraforaminal (E). **b**, **c** Representative cases of junctional and foraminal contrast distribution, respectively. Yellow arrows indicate the tip of the contrast spread. **b** The contrast extends along the oblique line within the junctional zone. **c** The contrast is clearly confined medial to the line within the foraminal zone

We first evaluated contrast distribution on anteroposterior radiographs in all three groups (FL, US, ACL) by adapting the CT-based classification proposed by Lagemann et al. to plain radiography. On each radiograph, we identified the lateral mass of the target vertebra and measured the most proximal location of contrast relative to that landmark. Using the anatomical definitions of Lagemann et al. [15], we defined three zones: any contrast lying entirely medial to the lateral mass was assigned to the foraminal zone (F); contrast extending laterally but remaining within 2 mm proximal or distal to the lateral mass was assigned to the junctional zone (J); and contrast extending more than 2 mm lateral to the lateral mass was assigned to the extraforaminal zone (E) (Fig. 5). We used radiographs for this initial assessment because, in our retrospective cohort, CT imaging was not available for the FL group. For the US and ACL groups only, we then confirmed contrast distribution on axial CT images using the original foraminal, junctional, and extraforaminal definitions of Lagemann et al. [15] (Fig. 6). A fellowship-trained orthopedic surgeon blinded to injection technique performed all CT-based classifications independently. This two-step approach allowed us to compare distribution patterns across all groups on radiographs and to verify precise intraforaminal delivery with CT in the ultrasound-guided cohorts.

### Sample size and statistical analysis

Continuous variables are reported as mean ± SD and categorical variables as counts with percentages. Group comparisons for continuous outcomes were made using one-way ANOVA followed by Tukey’s multiple comparison test. Categorical variables were compared using Fisher’s exact test. Two-tailed significance was defined as *P* < 0.05, and all primary analyses were performed in GraphPad Prism 10 (GraphPad Software, San Diego, CA). To confirm that our sample size of 114 provided sufficient power to detect the observed differences in needle tip distance, we performed a post hoc power calculation in G*Power 3.1. Using the observed group means and a pooled standard deviation of 1.0 with an alpha error probability of 0.05, the calculated effect size f was 3.44 and the achieved power was 1.0.

## Results

Of 114 patients (56 FL group, 25 US group, 33 ACL group), mean age, sex, height, body weight and BMI did not differ among groups (Table 1). One patient (3%) in the ACL group developed Horner syndrome (*P* = 0.31). No vascular or nerve injuries occurred in any group.

**Table 1:** Comparison of baseline characteristics by guidance technique. Data are presented as mean ± standard deviation or number (percentage) unless otherwise indicated

Comparison of baseline characteristics by guidance technique							
Variable	FL (*n* = 56)	US (*n* = 25)	ACL (*n* = 33)	95% CI (FL vs US)	95% CI (FL vs ACL)	95% CI (US vs ACL)	*P* value
Age (years)	55.0 ± 13.0	58.0 ± 11.6	61.2 ± 16.3	−10.9 to 4.9	−13.4 to 1.0	−11.9 to 5.5	0.12
Female	21 (37.5%)	16 (64%)	19 (57.6%)	–	–	–	0.13
Height (m)	1.65 ± 0.09	1.65 ± 0.08	1.67 ± 0.10	−0.05 to 0.05	−0.07 to 0.03	−0.08 to 0.04	0.58
Weight (kg)	65.7 ± 15.2	69.3 ± 11.4	66.3 ± 12.1	−11.4 to 4.2	−7.7 to 6.5	−5.5 to 11.6	0.53
BMI (kg/m^2^)	24.0 ± 4.2	25.6 ± 3.8	23.7 ± 2.8	−3.7 to 0.6	−1.7 to 2.2	−0.5 to 4.2	0.15

FL, fluoroscopy-guided group; US, conventional ultrasound-guided group; ACL, anterior compression lateral ultrasound-guided group; BMI, body mass index; CI, confidence interval

**Fig. 7:**
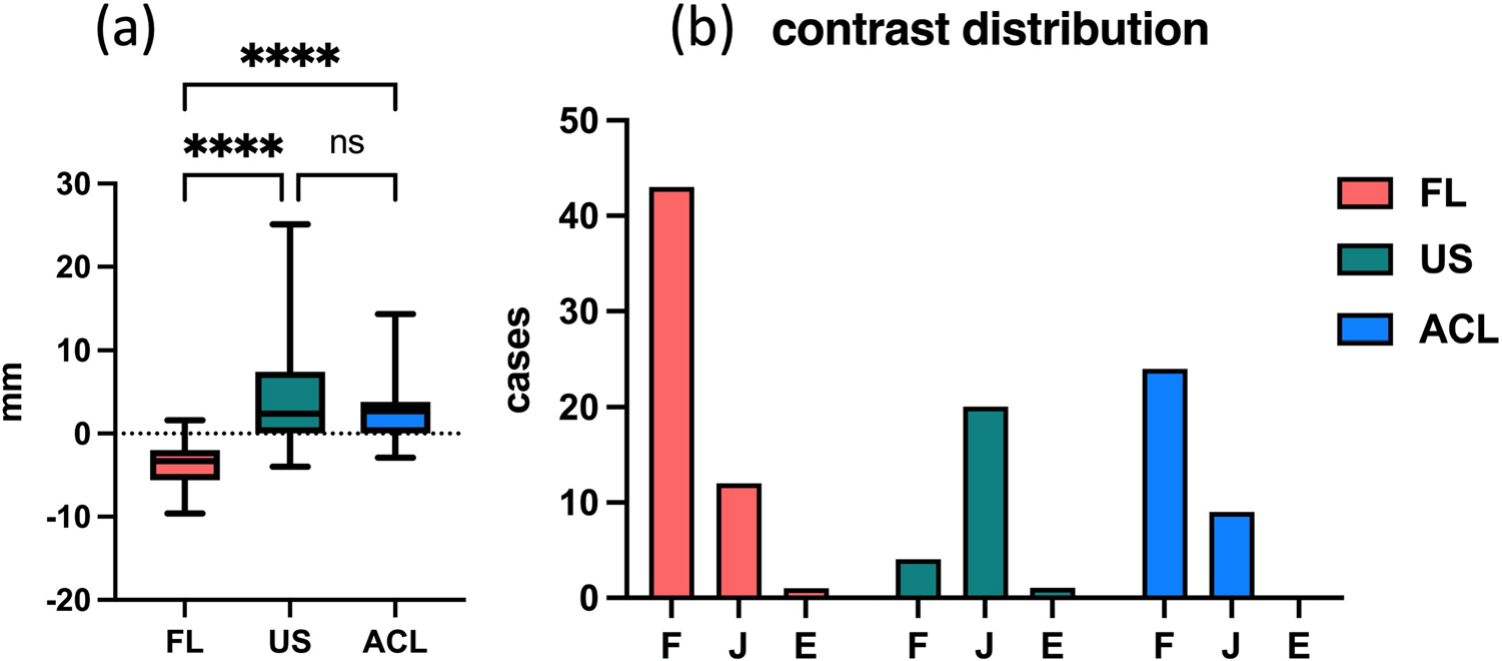
**a** Comparison of needle tip distance from the lateral mass and injectate spread distance across groups. **b** Distribution of contrast patterns by group. Foraminal distribution (F) was most common in the FL (76.8%) and ACL (72.7%) groups, while junctional (J) and extraforaminal (E) distributions were more frequent in the US group (*P* < 0.0001). FL: fluoroscopy, US: conventional ultrasound, ACL: anterior compression lateral ultrasound approach

Needle tip distance from the lateral mass varied by technique (*P* < 0.0001). Mean distance was –3.5 ± 2.6 mm in the FL group, 4.3 ± 6.8 mm in the US group, and 2.5 ± 3.9 mm in the ACL group. Compared with FL, the US group placed the needle tip 7.8 mm farther from the lateral mass (95% CI, 5.4–10.2 mm; *P* < 0.0001) and the ACL group placed it 6.0 mm farther (95% CI, 3.8–8.2 mm; *P* < 0.0001). There was no difference between US and ACL (mean difference, 1.8 mm; 95% CI, −0.9–4.4 mm; *P* = 0.27) (Fig. 7a). Injectate spread distance also differed among techniques (*P* < 0.0001). Mean spread was 15.9 ± 10.7 mm in the FL group, 30.8 ± 9.6 mm in the US group, and 25.9 ± 15.1 mm in the ACL group. Spread in the US group exceeded that in the FL group by 14.9 mm (95% CI, 8.1–21.7 mm; *P* < 0.0001) and in the ACL group by 10.0 mm (95% CI, 3.8–16.2 mm; *P* = 0.0007). The US and ACL groups did not differ (mean difference, 4.9 mm; 95% CI, −2.6–12.5 mm; *P* = 0.27).

In all patients, contrast distribution on anteroposterior radiographs differed significantly among groups (*P* < 0.0001). In the FL group, 76.8% of injections reached the radiographic foraminal zone (F) versus 16.0% in the US group and 72.7% in the ACL group. The junctional zone (J) appearances were seen in 21.4% of FL, 80.0% of US, and 27.3% of ACL injections, reflecting a distribution in ACL similar to FL and markedly different from US (Fig. 7b). In the CT-based subgroup (US n = 25, ACL *n* = 33), F occurred in four (16.0%) US versus 24 (72.7%) ACL injections and J in 20 (80.0%) US versus nine (27.3%) ACL injections (*P* < 0.0001), confirming that ACL significantly improves intraforaminal delivery, consistent with the radiographic anteroposterior findings for US and ACL.

## Discussion

In this retrospective cohort study, we directly compared three image-guided cervical selective nerve root block (SNRB) techniques (FL group, US group, and ACL group). The principal finding was that ACL achieved an intraforaminal (F) distribution of contrast in 72.7% of injections, statistically indistinguishable from the 76.8% attained with the current gold-standard FL group, yet more than fourfold higher than the 16.0% observed with the posterior US approach (*P* < 0.001). Since accurate intraforaminal delivery is believed to be anatomically desirable for effective treatment of radiculopathy, these data position ACL as a radiation-free alternative that preserves anatomical precision.

Multiple studies have highlighted the inherent limitations of the conventional posterior ultrasound-guided approach. Yamauchi and colleagues performed CT evaluations and found that in all cases, the injectate delivered via posterior ultrasound-guided cervical nerve root block spread exclusively to the extraforaminal region, with no evidence of epidural or intraforaminal penetration [24]. More recently, Ma et al. reported that only 9.5% of such blocks reached the epidural space, while the remainder failed to achieve foraminal distribution [7]. Our finding of a low intraforaminal distribution rate (16.0%) corroborates these results and underscores that posterior ultrasound-guided techniques frequently fall short of delivering medication into the clinically essential foraminal and epidural spaces. Together, the evidence indicates that anatomic barriers, such as the transverse process and articular pillar, impose a structural ceiling on targeting accuracy—one that operator experience alone is unlikely to overcome.

The ACL approach was conceived by transposing the CT-guided anterolateral transforaminal trajectory, long recognized for reliable needle placement, into ultrasound guidance [17, 25]. Built on this anatomical rationale, the technique aims to deliver injectate within or adjacent to the intervertebral foramen, that is, the compartment surrounding the symptomatic nerve root and dorsal root ganglion where periganglionic inflammation, conduction abnormalities, and microvascular changes concentrate [26, 27]. While this foraminal-proximal targeting is anatomically desirable, comparative studies report broadly similar short- to mid-term outcomes across guidance modalities despite differing spread patterns [6, 28]. Moreover, CT-fluoroscopic data suggest that exiting or extraforaminal needle tip positions may lower intravascular injection risk [15]. Accordingly, our rationale is anatomical rather than a claim of clinical superiority, and prospective studies correlating precise injectate location with standardized clinical outcomes are warranted.

The average craniocaudal spread distance was twofold greater with ultrasound-guided injections (US 30.8 mm, ACL 25.9 mm) than with FL (15.9 mm). Since FL needles terminate within the narrow osseous channel bordered by the pedicle and facet joint, injectate is largely confined. In contrast, both US paths traverse compliant fascia and loose areolar tissue before entering the foramen; the resulting lower outflow resistance permits broader distribution along the nerve sheath. A similar pattern was documented by Kang et al., who found that increasing injectate volume during posterior US SNRB produced preferential longitudinal rather than radial spread [14]. While greater spread may augment therapeutic coverage, its clinical significance requires prospective correlation with symptom relief and adverse effects. Given these considerations, the present imaging advantages should be interpreted as anatomical plausibility rather than established clinical superiority, pending prospective correlation with pain/function outcomes and re-intervention rates.

A key strength of the present study was our use of thin-slice CT as an objective endpoint for every participant in the ultrasound cohorts, removing reliance on indirect fluoroscopic landmarks; the sample size (*n* = 114) also yielded > 99% post hoc power to detect the observed between-group differences. Limitations included the retrospective, single-center design with potential selection bias and unmeasured confounding (for example, needle gauge and subtle technique variation) that could have affected contrast behavior. We assessed imaging surrogates rather than clinical outcomes, so correlations between imaging findings and clinical effectiveness cannot be inferred and should be tested prospectively with standardized pain, function, and re-intervention endpoints. Exclusion of patients without anthropometric data may limit generalizability. In the fluoroscopy cohort, contrast spread was evaluated on radiographs rather than CT, which may have limited cross-modality comparability. In addition, needle tip distance from the lateral mass was measured on anteroposterior radiographs without individual geometric scaling; differences in body habitus could therefore have introduced magnification bias. Since all imaging was acquired on the same equipment under a standardized protocol at a single center, systematic magnification differences between groups were likely small; nonetheless, future studies should incorporate external scaling markers or post-processing correction. Finally, all ACL procedures were performed by a single experienced surgeon, and we did not quantify reader agreement for image-based outcomes; future multi-operator studies with predefined learning-curve assessment and formal inter- and intra-rater metrics (κ/ICC) are warranted.

To establish the clinical value of ACL, a prospective randomized trial comparing ACL, FL, conventional US, and other emerging sonographic strategies is needed. Stratification by foraminal stenosis severity and injectate volume may reveal subgroups that benefit most from each technique. In addition, incorporation of color Doppler mapping to visualize vertebral artery position could further enhance ACL safety.

## Conclusions

The ACL approach to guided visualization achieves injectate distribution that matches the fluoroscopic technique while avoiding radiation exposure. It provides targeted intraforaminal delivery like the FL group and benefits from the broader soft tissue spread seen with ultrasound-guided methods. Prospective trials that measure pain relief and functional outcomes will be essential to confirm the clinical advantages of this approach.

## Data Availability

The datasets generated and analyzed during the current study are not publicly available due to ethical and privacy restrictions;however, they are available from the corresponding author upon reasonable request.
